# Arachidonic acid induces macrophage cell cycle arrest through the JNK signaling pathway

**DOI:** 10.1186/s12944-018-0673-0

**Published:** 2018-02-09

**Authors:** Ziying Shen, Yunqing Ma, Zhonghao Ji, Yang Hao, Xuan Yan, Yuan Zhong, Xiaochun Tang, Wenzhi Ren

**Affiliations:** Laboratory Animal Center, College of Animal Sciences, Jilin University, Xi’an Road, 5333#, Jilin, 130062 China

**Keywords:** Arachidonic acid, RAW264.7 cells, Cell cycle arrest, JNK signaling pathway, Forkhead box proteins

## Abstract

**Background:**

Arachidonic acid (AA) has potent pro-apoptotic effects on cancer cells at a low concentration and on macrophages at a very high concentration. However, the effects of AA on the macrophage cell cycle and related signaling pathways have not been fully investigated. Herein we aim to observe the effect of AA on macrophages cell cycle.

**Results:**

AA exposure reduced the viability and number of macrophages in a dose- and time-dependent manner. The reduction in RAW264.7 cell viability was not caused by apoptosis, as indicated by caspase-3 and activated caspase-3 detection. Further research illustrated that AA exposure induced RAW264.7 cell cycle arrested at S phase, and some cell cycle-regulated proteins were altered accordingly. Moreover, JNK signaling was stimulated by AA, and the stimulation was partially reversed by a JNK signaling inhibitor in accordance with cell cycle-related factors. In addition, nuclear and total Foxo1/3a and phosphorylated Foxo1/3a were elevated by AA in a dose- and time-dependent manner, and this elevation was suppressed by the JNK signaling inhibitor.

**Conclusion:**

Our study demonstrated that AA inhibits macrophage viability by inducing S phase cell cycle arrest. The JNK signaling pathway and the downstream FoxO transcription factors are involved in AA-induced RAW264.7 cell cycle arrest.

**Electronic supplementary material:**

The online version of this article (10.1186/s12944-018-0673-0) contains supplementary material, which is available to authorized users.

## Background

Arachidonic acid (AA), an omega-6 long-chain polyunsaturated fatty acid, which is a crucial membrane phospholipid in maintaining the elderly brain function and used as a supplement in infant dietary to promote brain development [1–3]. AA is a precursor which can respectively be metabolized by Cyclooxygenase (COX), lipoxygenase (LOX) and cytochome P450 (CYP450) to prostaglandins, leukotrienes and epoxyeicosatrienoic acids [4]. The in vivo metabolites of AA are a variety of proinflammatory eicosanoids that function in the inflammatory networks of the body and affect cells involved in acquired immunity [5, 6]. One of the COX metabolites, TXA2 is a potent vasoconstrictor which can induce an inflammatory vascular response by stimulating the vasculature to secrete proinflammatory cytokines and adhesion molecules, causing peripheral blood mononuclear cells (PBMCs) to aggregate in the inflammatory area [7–10]; one of the 5-LOX metabolites, LTB4 can upregulate the expression of CD36 (a macrophage scavenger receptor) and promote the uptake and accumulation of LDL and lipids, which facilitate the formation of foam cells [8, 11, 12].

Proinflammatory cytokines as a signal can activate the stress-activated protein kinases (also termed JNK) which control apoptosis and growth. The effect of activated JNK depends on the cell types and other signals triggered. FoxO proteins as a downstream of JNK [13–15] also participate in various cellular processes, including cell proliferation, cell cycle and apoptosis [15, 16].

Macrophages function in innate immune response are part of the mononuclear phagocyte system. Macrophages can release and transfer AA both in vitro and in vivo [17–19]. The phagocytic activity of macrophages is positively correlated with the concentration of AA, and high levels of AA induces other types of cell apoptosis [20]. These results indicate that macrophage activity may be influenced by the local AA concentration. However, how the macrophage cell cycle responds to AA remains unknown. We therefore explored the effect of AA on the macrophage cell cycle and the mechanisms involved in.

## Methods

### Reagents

Antibodies against β-actin, GADPH, caspase-3, activated caspase-3, cyclin D, CDK4, P53, P21, JNK(1/2), JNK2, SP600125 (inhibitor of JNK), Foxo1, Foxo3a, and p-Foxo1/3a and the Prestained Protein Molecular Weight Marker were purchased from Beyotime (China). Antibodies against cyclin E, CDK2, and Lamin B1, TNF-α, IL-2, COX-2 were purchased from Proteintech Group Inc. The p-JNK(1/2) and JNK1 antibody was purchased from Cell Signaling Technology (USA). DMSO, tween-20, KETO, NDGA, TPPU were purchased from Sigma(USA). ABT was purchased from Tocris(USA). PBS powder, TBS powder and nonfat milk powder were purchased from Boster (China).

### Cell culture and treatment

RAW264.7 cells were obtained from stocks preserved in the Biochemistry Laboratory of Jilin University and incubated in RPMI 1640 medium supplemented with 10% fetal bovine serum (FBS), 1% 100× penicillin-streptomycin solution and 1% 100× L-glutamine at 37 °C under 5% CO_2_. Prior to experiments, the cells were serum starved by a 12 h incubation in medium containing 0.5% FBS. All the reagents above were purchased from Gibco (USA). PBMCs separated from mice and New Zealand White rabbits were cultured in the same manner as the RAW264.7 cells.

### Cell viability and proliferation assay

Cells were plated in 96-well cultured plates at a density of 1 × 10^4^ cells per well and then treated with AA (Cayman Chemical, USA) at 0 μM (control group), 40 μM, 60 μM, or 80 μM for 12 h and 24 h after a 12 h starved.

According to the manufacturer’s instructions of the Cell Counting Kit 8 (DOJINDO, Japan), 10 μL of CCK8 reagent was added to each well. The plate was incubated at 37 °C for 1~ 3 h and then measured by a microplate reader at 450 nm. The data obtained from this experiment were analyzed and plotted.

Proliferation was detected by counting the number of cells after treatment with various concentrations of AA for 12 h. The original medium was replaced with 0.25% trypsin to obtain single cells, and the trypsin was then removed. After diluting the cell suspension to the appropriate proportion, the cells were counted with a cell counting plate, and the data were statistically analyzed.

### Cell cycle analysis

Cells were plated in 6-well plates at a density of 5 × 10^5^ cells per well and then treated with 0 μM (control group) AA, 40 μM AA, 60 μM AA or 80 μM AA for 12 h after being starved. The cells were digested with 0.25% trypsin, and cell clumps were dispersed to single cells. The cells were fixed in pre-cooled 70% ethanol and stored at − 20 °C overnight.

According to the instructions of the Cell Cycle and Apoptosis Analysis Kit (Beyotime), 300 μL of working DNA-binding propidium iodide solution was added to the cells, and then incubated the cells in a dark chamber at 37 °C for 30 min. After staining, the red fluorescence was detected at 488 nm by flow cytometry, and the data were analyzed and plotted.

### Western blot

Treated cells were washed twice with PBS and lysed in lysis buffer (Beyotime) with 1% PMSF (Beyotime) and 1% 100× phosphatase inhibitor cocktail (Applygen Technologies Inc., China) at 4 °C for 30 min. The mixture was transferred to a centrifuge tube and spun for 5 min at 12000 rpm in a cryogenic centrifuge. The supernatant was collected in a new centrifuge tube and stored at − 20 °C for subsequent experiments.

Nuclear and cytoplasmic protein fractions were obtained using a Nuclear and Cytoplasmic Protein Extraction Kit (Beyotime) according to the manufacturer’s instructions.

The concentration of the protein samples was measured by a BCA protein assay kit (Beyotime) to ensure that the amount of total protein used from each group was the same. The samples were separated by 8%~ 12% sodium dodecyl sulfate-polyacrylamide gel electrophoresis and then transferred to polyvinyl difluoride (PVDF) membranes. The membranes were blocked with TBST containing 5% (wt/vol) nonfat milk for 1.5 h at room temperature (RM) and then incubated at 4 °C overnight with primary antibodies. The membranes were washed with TBST thee times for 10 min by shaking and then incubated with the appropriate secondary antibody at RM for 1 h. The Pierce Enhanced Chemiluminescence (ECL) Plus Western Blotting Substrate (Thermo Scientific, USA) was used to detect the bound proteins after TBST washing to remove any unbound secondary antibody.

### Statistical analysis

The experimental data were presented as the mean ± SEM. The statistical analysis was performed using an unpaired t-test with GraphPad Prism software. *P* < 0.05 was considered to be statistically significant. *P < 0.05, **P < 0.01, ***P < 0.001. All figures in this study were obtained from at least three independent experiments with similar results.

## Results

### AA inhibits macrophage viability

**Fig. 1:**
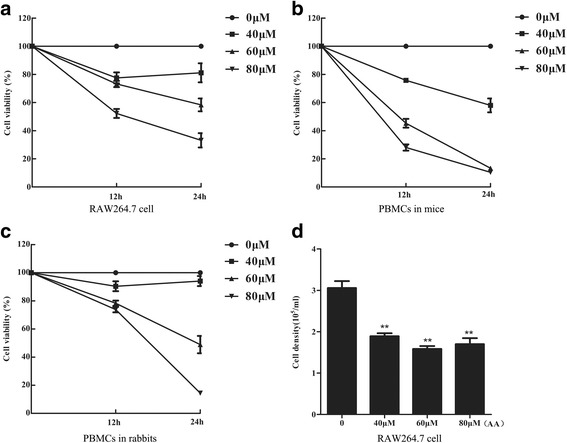
AA inhibits macrophage viability. **a** RAW264.7 cells were pre-treated with the indicated concentrations of AA for 12 h or 24 h. The cell viability was measured by CCK8 assay. **b** and **c**. PBMC-derived macrophages from mice and rabbits were pre-treated with the indicated concentrations of AA for 12 h or 24 h. The cell viability was measured by CCK8 assay. **d** RAW264.7 cells were pre-treated with the indicated doses of AA for 12 h, and the cell numbers were counted. *N* = 4

The effects of AA on macrophage viability were investigated in RAW264.7 cells and PBMC-derived macrophages from mice and rabbits. The cells were incubated with different doses of AA, and CCK8 assays were performed after 12 h and 24 h of treatment. AA significantly inhibited macrophage viability within 12 h in a dose-dependent manner, and slightly more inhibition was observed between 12 h to 24 h (Fig. 1a-c). A cell counting analysis of RAW264.7 cells also demonstrated the significant inhibitory effects of AA (Fig. 1d). The data showed that AA suppressed macrophage viability at certain concentrations.

### AA induces macrophage cell cycle arrest at S phase

**Fig. 2:**
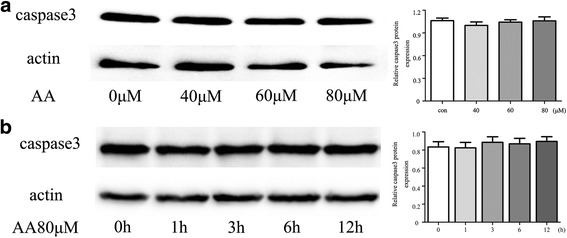
Caspase-3 expression in RAW264.7 cells. **a** RAW264.7 cells were pre-treated with AA for 12 h at the indicated concentrations. **b** RAW264.7 cells were pre-treated with 80 μM AA for the indicated length of time. Total protein was immunoblotted with Caspase-3 antibody. The figure containes the densitometric quantification of relative protein expression

**Fig. 3:**
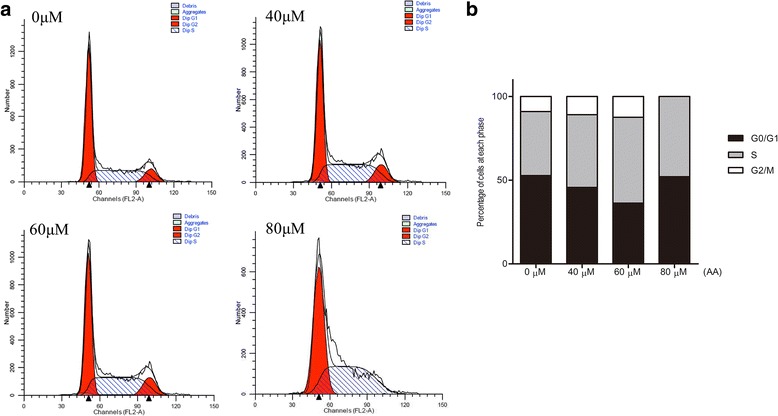
AA induces macrophage cell cycle arrest. **a** RAW264.7 cells were pre-treated with AA for 12 h at the indicated concentrations, and flow cytometry was performed. **b** Statistical analysis of the flow cytometry results by GraphPad Prism

Decreased cell viability may be caused by apoptosis. Caspase-3 and activated caspase-3 were detected in RAW264.7 cells incubated with different doses of AA (0 μM, 40 μM, 60 μM, 80 μM) for 12 h. However, activated caspase-3 (17 kDa) was not detected in any of the indicated conditions (Fig. 2a-b). Further flow cytometry results showed that the proportion of RAW264.7 cells in S phase increased upon treatment with AA at the indicated concentrations (Fig. 3a-b). The data illustrated that AA inhibits macrophage viability by inducing cell cycle arrest.

### AA regulates cell cycle-related genes in RAW264.7 cells

**Fig. 4:**
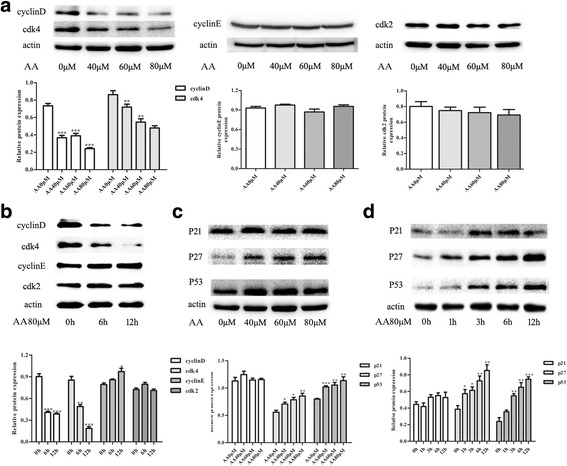
AA regulates cell cycle-related genes in RAW264.7 cells. Total proteins were immunoblotted with the indicated antibodies. **a** and **c** RAW264.7 cells were pre-treated with AA at the indicated doses for 12 h. **b** and **d** RAW264.7 cells were pre-treated with 80 μM AA for the indicated length of time. The figure containes the densitometric quantification of relative protein expression

The expression of cell cycle-related genes was detected in RAW264.7 cells after incubation with different AA doses and for different lengths of time. Western blotting demonstrated that the expression of cyclinD and CDK4 decreased significantly after treatment with the indicated AA doses and for the indicated length of time; however, cyclinE and CDK2 did not exhibit clear alterations after AA exposure (Fig. 4a-b). Furthermore, the cyclin-dependent kinase inhibitors p53 and p27 increased upon AA treatment in a dose- and time-dependent manner (Fig. 4c-d). The results confirmed that AA inhibits macrophage viability by regulating cell cycle arrest.

### JNK signaling pathway is involved in AA-induced cell cycle arrest in RAW264.7 cells

**Fig. 5:**
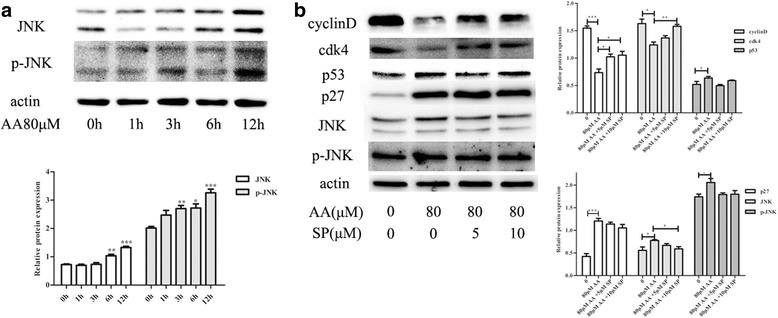
JNK signaling pathway is involved in AA-induced cell cycle arrest. Total proteins were immunoblotted with the indicated antibodies. **a** RAW264.7 cells were pre-treated with 80 μM AA for the indicated length of time. **b** RAW264.7 cells were pre-treated with SP6000125 for 1 h and then co-incubated with AA for 12 h. The figure containes the densitometric quantification of relative protein expression

The JNK signaling pathway is a subset of the MAPK pathway and plays important roles in cell cycle and inflammation. RAW264.7 cells were incubated with 80 μM AA for the indicated amounts of time, and the expression of both total JNK1/2 protein and phosphorylated JNK1/2 increased significantly (Fig. 5a). JNK pathway inhibitor (SP6000125) partially reversed the AA-mediated increase in p53 and decrease in cyclinD and CDK4 in accordance with the suppression of JNK but did not alter p27 expression (Fig. 5b). These results showed that activation of JNK signaling pathway is involved in AA-induced cell cycle arrest.

### AA-induced JNK signaling pathway activate FoxOs in RAW264.7 cells

**Fig. 6:**
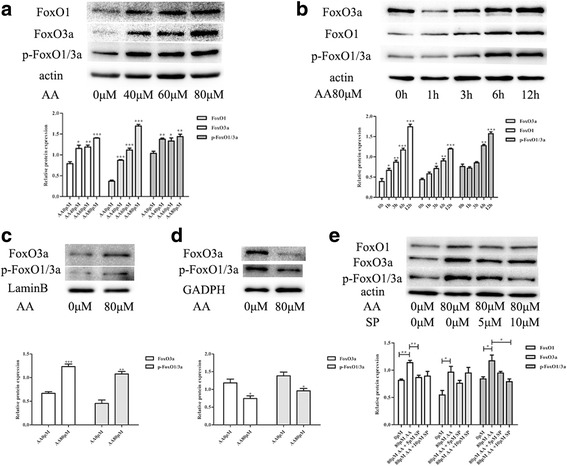
AA-induced JNK signaling pathway activate FoxOs in RAW264.7 cells. Proteins were immunoblotted with the indicated antibodies. **a** RAW264.7 cells were pre-treated with the indicated concentrations of AA for 12 h. **b** RAW264.7 cells were pre-treated with 80 μM AA for the indicated length of time. **c** and **d** RAW264.7 cells were pre-treated with 80 μM AA for 12 h, and nuclear and cytoplasmic proteins were extracted. **e** RAW264.7 cells were pre-treated with SP6000125 for 1 h and then co-incubated with AA for 12 h. The figure containes the densitometric quantification of relative protein expression

Forkhead (FoxO) transcription factors are associated with cell proliferation, differentiation, apoptosis and the cell cycle. The expression of FoxO proteins was detected by western blot in RAW264.7 cells treated with different AA doses for different amounts of time, and the effect of JNK inhibition on the expression of FoxO proteins was also analyzed. AA significantly induced the protein expression of intracellular FoxO1 and FoxO3a and their phosphorylated forms (Fig. 6a, b). After treatment with 80 μM AA, the expression of intracellular FoxO3a initially decreased and gradually increased with incubation time (Fig. 6b). Further research demonstrated that the FoxO1/3a and phosphorylated FoxO1/3a induced by AA were primarily located in the nucleus (Fig. 6c, d). The AA-mediated induction of FoxO proteins was partially reversed by the JNK inhibitor SP6000125 (Fig. 6e).

## Discussion

Arachidonic acid, a n-6 polyunsaturated fatty acid, has a number of biological functions, is located on the cell membrane and plays a key role in maintaining membrane function and structure [21, 22]. Due to membrane fluidity, free AA participates in the delivery of intracellular signals [23, 24], and its metabolites prostaglandins, leukotrienes and thomboxanes are involved in many AA-mediated functions [11, 25], such as inflammatory processes [26], cancer [27] and vascular disease [28]. Many reports have shown that AA has potent pro-apoptotic effects on cancer cells [29]. Macrophages are important immune cells involved in both innate and adaptive immunity [30, 31]; however, there is little information regarding the effects of AA on macrophages. In this study, the macrophage model cell line RAW264.7 and PBMC-derived macrophages from mice and rabbits were used to investigate the effects of AA on macrophage viability and the mechanisms involved. Macrophage activity was inhibited by AA in a dose- and time-dependent manner, and treatment with 80 μM AA for 12 h was selected for use in subsequent experiments.

Apoptosis and cell cycle arrest are two possible causes of cell growth inhibition. Although AA induced apoptosis of Mouse Follicular Granulosa Cells [14], brain endothelial cell [32], AS-30D cells [33], our results regarding the crucial enzymes activated caspase-3 and cleaved PARP (data not shown) indicated that AA did not promote RAW264.7 cell apoptosis. Further results demonstrated that RAW264.7 cells were arrested at S phase upon incubation with the indicated doses of AA. CyclinD and CDK4 were significantly inhibited by AA, but cyclinE and CDK2 were not obviously altered by AA at any dose or incubation time. Both of the cyclinD-CDK4 complex and cyclinE-CDK2 complex can promote G1 to S phase transition. Besides, p27 and p21 can inhibit CDK4 and destroy the combination of the complex. Accordance with the decreased cyclin D and CDK4, the increased expression of p27 can affect the enzymes for DNA duplicate [34, 35]. Figure 3b shows the S phase is generally increased by AA until the cell growth arrest at S phase. Previous reports indicated that a high concentration of AA (200 μM) causes apoptosis [29, 36]. In our study, the concentration of AA used was between 0~ 80 μM. The cell cycle-related proteins affected by AA may counterbalance each other to avoid apoptosis.

The JNK signaling pathway is involved in the regulation of cell proliferation, differentiation and apoptosis [37–40]. It also participates in the cancer cell cycle arrest caused by various stimuli [41–43]. In our study, both total JNK (1/2) and phosphorylated JNK (1/2) were increased significantly by AA, and this elevation could be suppressed by a JNK inhibitor, which also altered the expression of cell cycle-related proteins. FoxO transcription factors (FoxO1, FoxO3a, FoxO4 and FoxO6), which are downstream of the JNK signaling pathway, are important transcriptional regulators of target genes like Cyclin D, cyclin E, p21 and p27 that function in cell proliferation, differentiation and the cell cycle [15, 44–49]. We observed that total FoxO1, FoxO3a and phosphorylated FoxO1/3a were elevated upon AA-induced RAW264.7 cell cycle arrest. The increase in total FoxO1, FoxO3a and phosphorylated FoxO1/3a was reduced partially by the JNK signaling pathway inhibitor. JNK/FoxOs regulate the AA-induced RAW264.7 cell cycle arrest and participates in the counterbalance between cell proliferation and apoptosis.

AA is a precursor which can respectively be metabolized by Cyclooxygenase (COX), lipoxygenase (LOX) and cytochome P450 (CYP450) to prostaglandins, leukotrienes and epoxyeicosatrienoic acids. Cytochome P450 mainly metabolizes AA to 20-hydroxyeicosatetraenoic acid (20-HETE) and epoxyeicosatrienoic acids (EETs) which can be converted to dihydroxyiecosatrienoic acids (DHETs) by sEH [50, 51]. The LOX-inhibitor NDGA-a general inhibitor of LOXs, ABT which inhibits 20-HETE synthesis and TPPU which is a potent sEH inhibitor can alleviate the inhibition trend of RAW264.7 induced by AA, but the suppression still exists while the COX-inhibitor KETO aggravates the inhibition trend. Above results indicate that the metabolites are factors counterbalance each other to mediate the AA effect on RAW264.7 cells. There are some additional research based on the datas aboved but not showed in this study which also can prove our results in other ways (Additional files 1, 2, 3, 4, 5, 6: Figure S1-6). However, the detailed mechanism requires further investigation.

## Conclusion

In conclusion, our study demonstrated that AA inhibits macrophage viability by inducing S phase cell cycle arrest. The JNK signaling pathway and the downstream FoxO transcription factors are involved in AA-induced RAW264.7 cell cycle arrest.

## Additional files

Additional file 1: **Figure S1.** The expression of JNK1 and JNK2 respectively affected by AA. A-B, RAW264.7 cells were treated by indicated doses of AA for 12 h. The figure containes the densitometric quantification of relative protein expression.  (TIFF 183 kb)

Additional file 2: **Figure S2.** SP006125 inhibits the expression of COX-2, TNF-α and IL-2. RAW264.7 cells were pre-treated with indicated doses of SP000125 for 1 h and then co-incubated with 80 μM AA for 12 h. The figure containes the densitometric quantification of relative protein expression. (TIFF 170 kb)

Additional file 3: **Figure S3.** Effect of AA metabolism inhabitor on RAW264.7 cell viability. RAW264.7 cells were treated with the indicated concentrations of KETO(A), NDGA(B), ABT(C) and TPPU(D) for 12 h. The cell viability was measured by CCK8 assay. (TIFF 3794 kb)

Additional file 4: **Figure S4.** Effect of AA metabolism inhabitor and AA used together on RAW264.7 cell viability. A. RAW264.7 cells were pre-treated with 5 μM KETO for 1 h and then co-incubated with indicated doses of AA for 12 h. The cell viability was measured by CCK8 assay. B-D, the concentration of NDGA is 3 μM, ABT is 1 mM and TPPU is 1 μM. (TIFF 10197 kb)

Additional file 5: **Figure S5.** Fatty acid EPA and LA affect the viability of RAW364.7 cells. A. RAW264.7 cells were pre-treated with the indicated concentrations of EPA for 12 h or 24 h. The cell viability was measured by CCK8 assay. B RAW264.7 cells were pre-treated with the indicated concentrations of LA for 12 h or 24 h. The cell viability was measured by CCK8 assay. (TIFF 1776 kb)

Additional file 6: **Figure S6.** The CCK8 result of 0.1%DMSO as AA dilution on RAW364.7 cells. (TIFF 327 kb)

## Abbreviations

ABT: 1H–Benzotriazol-1-amine
actin: β-actin
FoxO: forkhead transcription factors
h: hours
JNK: c-Jun N-terminal kinase
KETO: Ketoprofen
NDGA: Nordihydroguaiaretic acid
PBMCs: Peripheral blood mononuclear cells
PBS: Phosphate-buffered saline
p-FoxO: phosphorylated forkhead transcription factors
p-JNK: phosphorylated JNK
PMSF: Phenylmethanesulfonylfluoride
PUFA: Polyunsaturated fatty acid AA arachidonic acid
PVDF: Polyvinyl difluoride
RM: Room temperature
SP: JNK inhibitors (SP600125)
TPPU: N-[1-(1-oxopropyl)-4-piperidinyl]-N0-[4-(trifluoromethoxy)phenyl)-urea
