# Supplementary figures and images for: Arachidonic acid induces macrophage cell cycle arrest through the JNK signaling pathway
Source: Lipids Health Dis. 2018 Feb 9;17:26. doi: 10.1186/s12944-018-0673-0 (PMC5807765; doi:10.1186/s12944-018-0673-0)

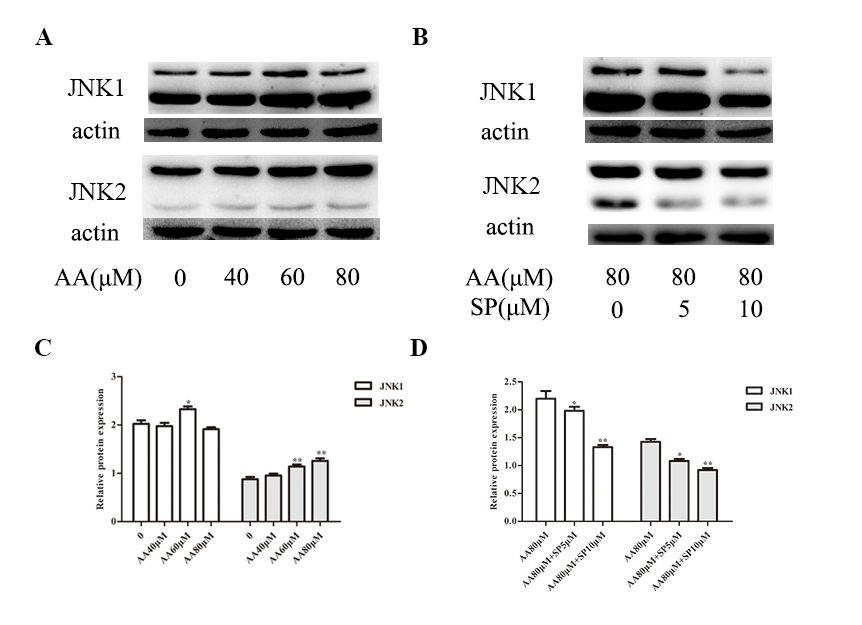

Supplement: Supplementary file 1 — Figure S1. The expression of JNK1 and JNK2 respectively affected by AA. A-B, RAW264.7 cells were treated by indicated doses of AA for 12 h. The figure containes the densitometric quantification of relative protein expression. (TIFF 183 kb) [file 12944_2018_673_MOESM1_ESM.tif]

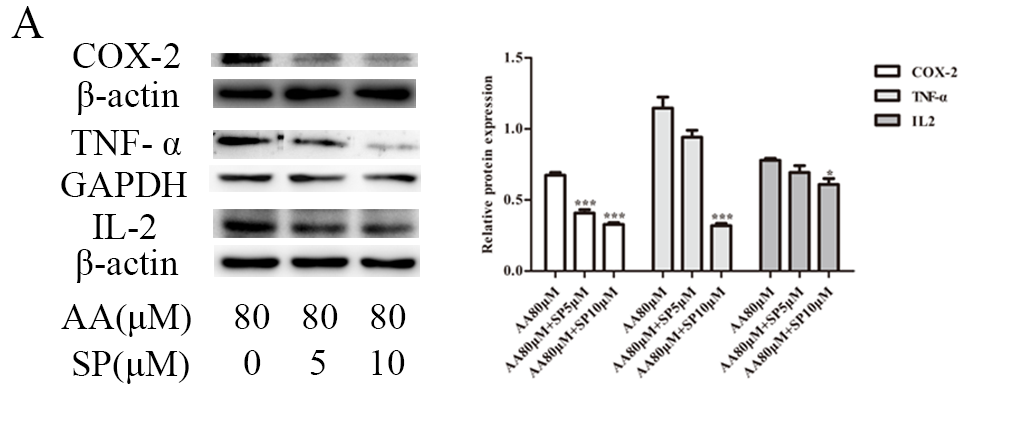

Supplement: Supplementary file 2 — Figure S2. SP006125 inhibits the expression of COX-2, TNF-α and IL-2. RAW264.7 cells were pre-treated with indicated doses of SP000125 for 1 h and then co-incubated with 80 μM AA for 12 h. The figure containes the densitometric quantification of relative protein expression. (TIFF 170 kb) [file 12944_2018_673_MOESM2_ESM.tif]

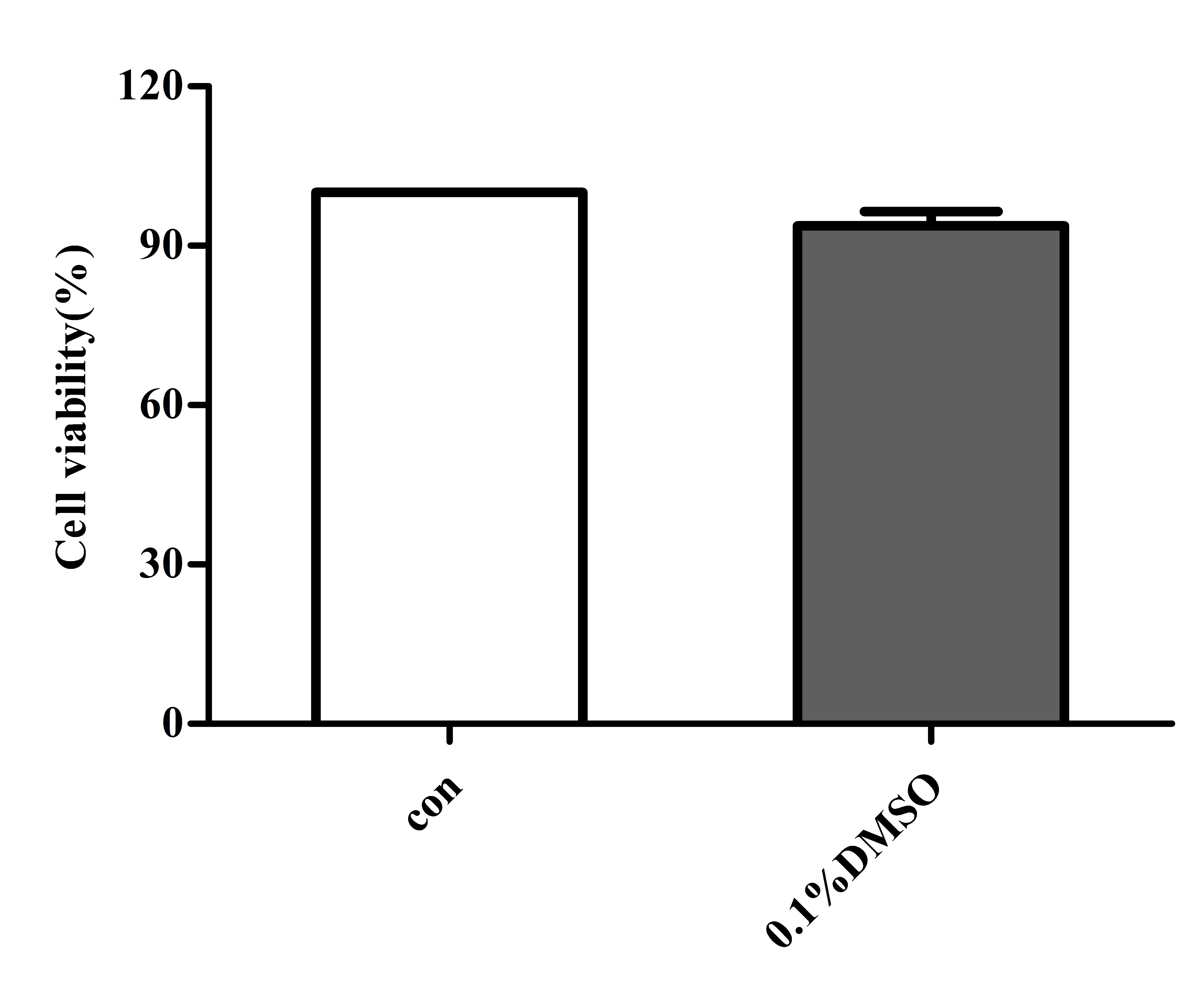

Supplement: Supplementary file 6 — Figure S6. The CCK8 result of 0.1%DMSO as AA dilution on RAW364.7 cells. (TIFF 327 kb) [file 12944_2018_673_MOESM6_ESM.tif]
